# Successful Treatment of a Critically Ill COVID-19 Patient Using Continuous Renal Replacement Therapy With Enhanced Cytokine Removal and Tocilizumab: A Case Report

**DOI:** 10.3389/fmed.2021.649583

**Published:** 2021-06-07

**Authors:** Thomas Tao-Min Huang, Ying-Chun Chien, Chih-Hsien Wang, Sui-Yuan Chang, Jann-Tay Wang, Song-Chou Hsieh, Yu-Chang Yeh, Shih-Chi Ku, Chong-Jen Yu, Bor-Luen Chiang, Shan-Chwen Chang, Ashita Tolwani

**Affiliations:** ^1^Department of Internal Medicine, National Taiwan University Hospital and College of Medicine, Taipei, Taiwan; ^2^Department of Surgery, National Taiwan University Hospital and College of Medicine, Taipei, Taiwan; ^3^Department of Clinical Laboratory Sciences and Medical Biotechnology, National Taiwan University College of Medicine, Taipei, Taiwan; ^4^Department of Anesthesiology, National Taiwan University Hospital and College of Medicine, Taipei, Taiwan; ^5^Department of Pediatrics, National Taiwan University Hospital and College of Medicine, National Taiwan University, Taipei, Taiwan; ^6^Division of Nephrology, University of Alabama, Birmingham, AL, United States

**Keywords:** COVID-19, cytokine release syndrome, extracorporeal membrane oxygenation, continuous renal replacement therapy, tocilizumab

## Abstract

The COVID-19 pandemic has caused multiple deaths worldwide. Since no specific therapies are currently available, treatment for critically ill patients with COVID-19 is supportive. The most severe patients need sustained life support for recovery. We herein describe the course of a critically ill COVID-19 patient with multi-organ failure, including acute respiratory failure, acute kidney injury, and fulminant cytokine release syndrome (CRS), who required mechanical ventilation and extracorporeal membrane oxygenation support. This patient with a predicted high mortality risk was successfully managed with a careful strategy of oxygenation, uremic toxin removal, hemodynamic support, and most importantly, cytokine-targeted intervention for CRS, including cytokine/endotoxin removal, anti-cytokine therapy, and immune modulation. Comprehensive cytokine data, CRS parameters, and biochemical data of extracorporeal removal were provided to strengthen the rationale of this strategy. In this report, we demonstrate that timely combined hemoperfusion with cytokine adsorptive capacity and anti-cytokine therapy can successfully treat COVID-19 patients with fulminant CRS. It also highlights the importance of implementing cytokine-targeted therapy for severe COVID-19 guided by the precise measurement of disease activity.

## Introduction

The emerging and rapid transmission of a novel coronavirus, severe acute respiratory syndrome coronavirus 2 (SARS-CoV-2), with its associated syndrome, coronavirus disease (COVID-19), has spread worldwide after its outbreak in Wuhan, China in late 2019 (1). Although most patients may be asymptomatic, patients with advanced ages or multiple comorbidities can develop

fatal illnesses and rapidly succumb to them. It is estimated that 2–10% of the patients may develop critical illness and may need intensive care and advanced life support (1–5). The associated pathophysiology is diverse and might range from transient organ dysfunction to deadly multi-organ failure. The organs involved include the cardiovascular system, kidneys, lungs, hematologic system, and immune system. Severely ill patients may have cytokine release syndrome (CRS) (6), presenting with refractory hypotension, hemophagocytic lymphohistiocytosis (7), anti-phospholipid activity (8), and kidney damage (9). Since effective antiviral therapy is not available at this time; life-supportive measures and effective complication management are pivotal measures for patient survival. We report herein a successful treatment strategy using cytokine-targeted therapy, including CRS management and extracorporeal cytokine removal, in a critically ill COVID-19 patient with a devastating clinical course.

## Case Description

A 53-year-old man with a history of colon cancer and bladder cancer with disease-free status for more than 5 years developed watery diarrhea 3 d after close contact with a confirmed COVID-19 patient. Fever (body temperature, 37.7°C), general malaise, myalgia, and poor appetite prompted him to seek medical advice on the 11th day, and he was admitted to a regional hospital. The hemogram was normal (white blood cell count: 4,900/μL, hemoglobin concentration: 16.6 g/dL, and platelet count: 203 k/μL). The aspartate aminotransferase (AST) and alanine aminotransferase (ALT) levels were 44 and 45 U/L, respectively. There was an elevation in the C-reactive protein (CRP) level (12.5 mg/dL), although the procalcitonin level was within normal limits (0.15 ng/mL). Polymerase chain reaction (PCR) from a nasal swab for SARS-CoV-2 was positive. Moxifloxacin (400 mg daily) and hydroxychloroquine were administered.

**Figure 1:**
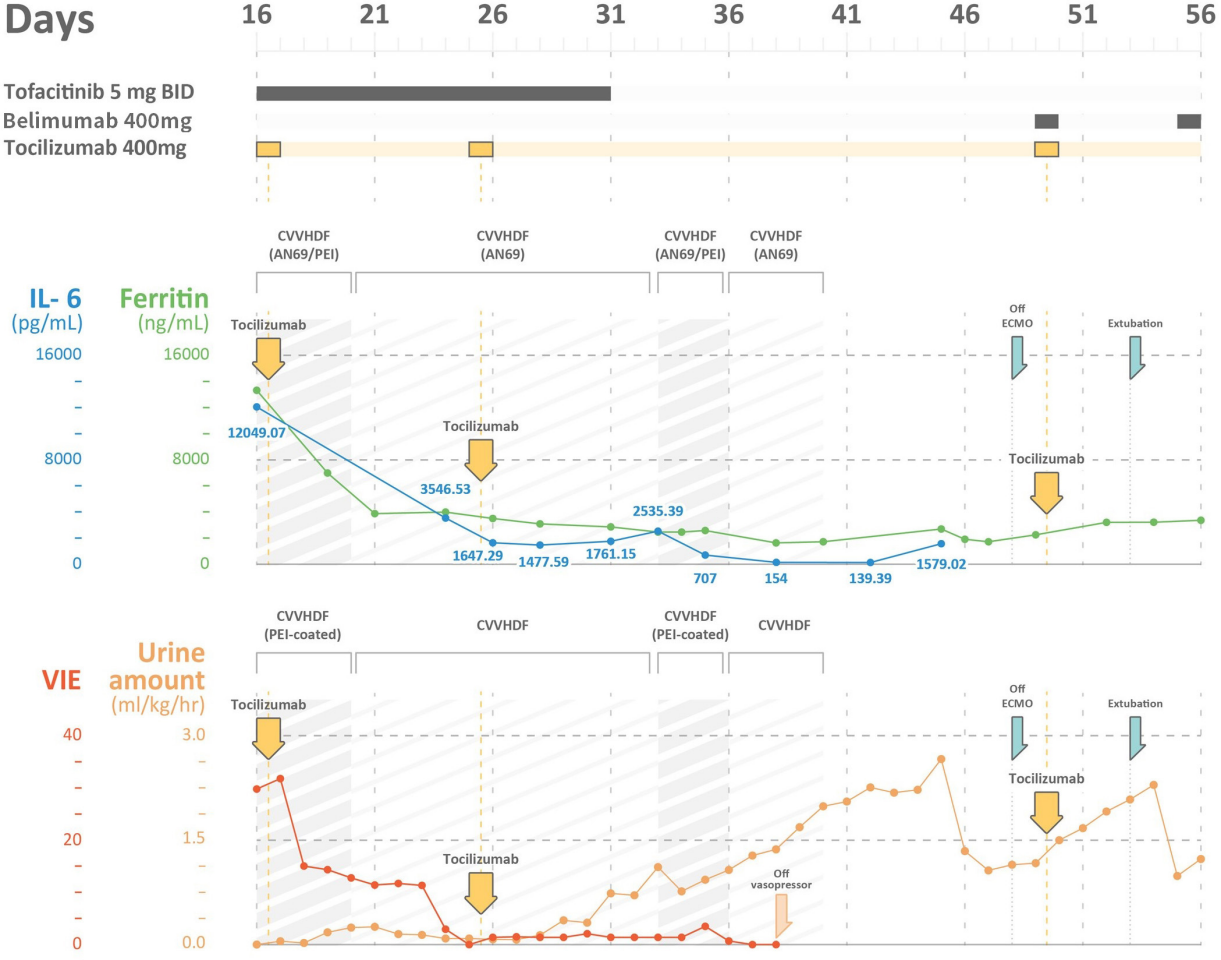
The time course of ferritin levels, IL-6 levels, vasoactive-inotropic equivalent (VIE) scores, and urine amount in a severe COVID-19 patient with multi-organ failures. IL-6, interleukin 6; VIE, vasoactive-inotropic equivalent; CVVHDF, continuous venovenous hemodiafiltration; AN69ST/PEI: hemofilter with AN69 and polyethyleneimine.

**Figure 2:**
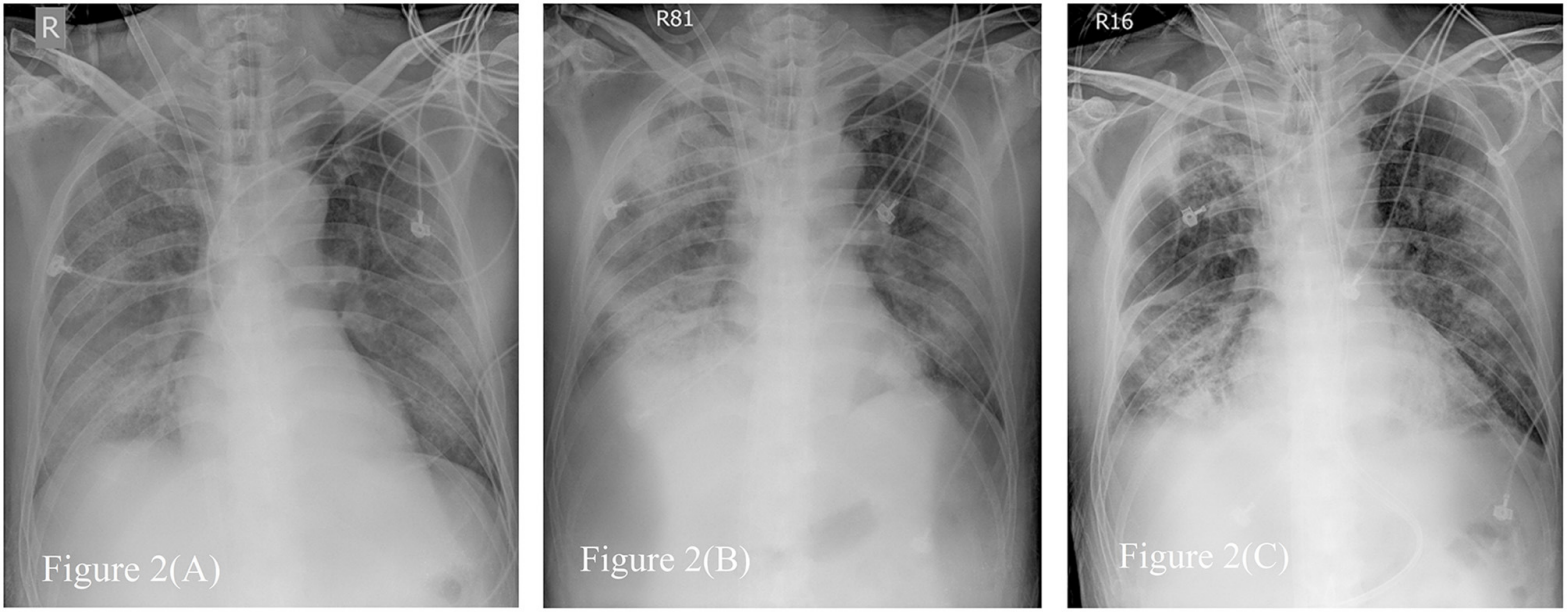
Serial Chest radiographs of the patient. **(A)** The film showing diffuse alveolar process over bilateral lung field, especially right lower lung field just after extracorporeal membrane oxygenator (ECMO setup). **(B)** The film revealing consolidation over both lungs when first time we tried to wean from ECMO. **(C)** The film on the day before removing ECMO, demonstrating fibrotic change as lung filed in **(B)**.

On day 16, he developed respiratory distress with an escalation of his oxygen needs from nasal prongs to a non-rebreathing mask. A chest radiograph revealed rapid progression of pulmonary infiltrates (**Figure 2A**). He underwent tracheal intubation for severe hypoxemia with an arterial oxygen partial pressure to fractional inspired oxygen ratio of 109 mmHg. In addition, he had lactic acidosis (lactic acid level 2.3 mg/dL), rhabdomyolysis (creatinine kinase level 261 U/L), high CRP level (14.6 mg/dL), and hyperferritinemia (ferritin level 2,957 ng/mL). His urine volume was ~840 mL in 24 h, and the renal reserve was adequate with a serum creatinine level of 1.1 mg/dL. With this presentation, he was transferred to our intensive care unit (ICU) for advanced life support (Figure 1).

## Disease Assessment

### Clinical Parameters and SARS-CoV-2 Detection

Acute disease severity was assessed using Acute Physiologic Assessment and Chronic Health Evaluation (APACHE) II scores collected on ICU admission and the next day of ICU stay (10). Sequential organ failure assessment (SOFA) scores were collected daily (11). Vasoactive-Inotropic Equivalent (VIE) scores were calculated from the infusion rates of vasopressors and inotropes and automatically summed up in the computerized database (12). Acute kidney injury was defined using the Kidney Disease: Improving Global Outcomes 2012 criteria (13). We also calculated the kinetic glomerular filtration rate (GFR), which forecasts future renal dysfunction derived from two consecutive days' serum creatinine data (14). Collection and detection of SARS-CoV-2 from biological samples were compliant with the World Health Organization guidance (15). Sputum, nasopharyngeal swabs, and oropharyngeal swabs were obtained for real-time PCR (RT-PCR). We determined the SARS-CoV-2 viral loads in each biological sample.

### Continuous Renal Replacement Therapy

The indication for continuous renal replacement therapy (CRRT) was oliguria for more than 12 h, with clinical evidence of fluid overload and hemodynamic compromise. We used a standard CRRT machine (Plasmaflex^Ⓡ^, Baxter^Ⓡ^, France) with either a standard AN69 based hemofilter (M150, Baxter^Ⓡ^, France) or a specialized hemofilter composed of AN69 and polyethyleneimine (AN69ST/PEI, oXiris^Ⓡ^, Baxter^Ⓡ^ France) used for increased clearance of cytokines and endotoxins in patients with sepsis (16). The CRRT prescription was compatible with the current standard (13). We used continuous veno-venous hemodiafiltration (CVVHDF) with a dialysate flow of 10–15 mL/kg/h along with a filtration flow of 15–20 mL/kg/h via a 14 Fr-uncuffed tunneled catheter. To decrease filtration fraction and prolong circuit lives, we chose CVVHDF as the main treatment modality. The filtration fraction was set to below 20% to avoid pre-mature dysfunction of the circuit. The AN69ST/PEI hemofilter with increased adsorptive capacity for cytokines and endotoxins has been emergently approved by the United States in response to the COVID-19 pandemic under EUA200164 and has been granted permission for use in Taiwan in recent years. We set up CRRT with an AN69ST/PEI along with heparin or regional citrate anticoagulation (RCA) (17). We targeted the activated prothrombin time around 50–70 s while using heparin and targeted a post-filter ionized calcium around 0.3–0.45 mmol/L while using RCA (18).

### Veno-Venous Extracorporeal Membrane Oxygenation Life Support

The indication for veno-venous extracorporeal membrane oxygenation (VV-ECMO) support was refractory hypoxemia. We cannulated the patient via the right internal jugular and femoral vein using the cut-down method. The VV-ECMO comprised a circuit with heparin-bound surfaces, an oxygenator (Affinity NT, Medtronic), a centrifugal pump (BPX-80 Bio-Pump Plus, Medtronic, Anaheim, CA, USA), and an oxygen-air blender (Model 3500 CP-G gas mixer, Sechrist, Anaheim, CA, USA) (19).

### Cytokine Measurement

We used the cytometric bead array (CBA) method to determine cytokine levels (20). Approximately 5 mL peripheral blood samples were obtained from patients at each time point. Plasma was isolated after centrifugation at 1,500 rpm for 10 min. The concentrations of cytokines (interleukin [IL]-2, IL-4, IL-6, IL-10, IL-17, interferon-γ, and tumor necrosis factor in plasma were determined using cytometric bead array (CBA; BD Biosciences), according to the manufacturer's protocol. Briefly, 350 μL samples were subjected to analysis in duplicate using the CBA kit on BD caliber cytometry. The concentrations of cytokines in culture supernatants were quantified using FCAP Array software v3.0 (20). We determined cytokine levels in samples collected from plasma and CRRT effluent. Plasma cytokine levels were checked at three-time points (before CRRT, hour 12, and 24) to determine the number of total cytokines removed using the area under the curve method. Because it was impossible to check the adsorptive capacity of AN69ST/PEI hemofilter, we checked cytokine levels from the CRRT effluent collected in the first 24 h on CRRT with the AN69ST/PEI hemofilter. The differences between these two values could approximate the number of cytokines absorbed by the hemofilter.

### Medications for Immune Modulation

The patient was prescribed tocilizumab, tofacitinib, hydroxychloroquine, and belimumab during his hospital course. Tocilizumab acts as an IL-6 receptor blockade and has been approved for rheumatoid arthritis, giant cell arteritis, and chimeric antigen receptor (CAR) T cell-induced severe or life-threatening cytokine release syndrome (21). Tofacitinib is an inhibitor of Janus kinase (JAKs) 1 and 3. It also has partial selectivity to JAK 2. Tofacitinib may suppress pro-inflammatory signaling including IL-6. It has not been tested in COVID-19 patients for cytokine blockade (22). Hydroxychloroquine is an anti-malarial drug and *in vitro* study confirmed its ability to inhibit SARS-CoV-2 (23). Antiphospholipid activities may be detected in COVID-19 patients. The mechanism of belimumab is to inhibit the binding of soluble circulating B lymphocyte stimulator to surface ligands of B cells, which may downregulate the anti-phospholipid activities (24).

## Results

Upon arrival to the ICU, the patient experienced rapid deterioration of oxygenation and hemodynamics. The body temperature was high (38.3°C), arterial blood pressure was low (79/51 mmHg), and he had tachycardia (heart rate, 136 bpm). He needed vasopressors with a VIE of 29.80: norepinephrine at 0.26 mcg/kg/min, and dopamine at 3.7 μg/kg/min were administered to maintain adequate mean artery pressure. The APACHE II score was 40 (corresponding to an estimated mortality rate of 85%), and the SOFA score was 17. There was a severe acute kidney injury (AKI) with an absence of urine output for more than 12 h and an increase in creatinine level to 3.1 mg/dL. The kinetic GFR was calculated to be 0 mL/min (14). Urinalysis showed dysmorphic red blood cells (RBCs) and renal tubular cells (RTCs), which indicated glomerulonephritis and acute tubular necrosis. A VV-ECMO circuit was promptly set for refractory hypoxemia (2). His laboratory values indicated a relative lymphopenia (lymphocyte percentage: 6.1%, with an absolute count of 722/μL). The ferritin level increased to 13,317 ng/mL, and the triglyceride level was 446 mg/dL. The AST level was 121 U/L, along with an ALT level of 47 U/L. The cytokine profiles displayed an extremely high IL-6 level (12,049.07 pg/mL). A rapidly evolving CRS was likely. In addition, with rapidly elevating procalcitonin levels (from 0.15 to >100 pg/mL), ceftazidime and levofloxacin were prescribed empirically for concern of a superimposed bacterial infection, although the bacterial cultures did not yield until discharge. CRRT with an AN69ST/PEI hemofilter was delivered using a separate uncuffed tunneled catheter. Intravenous immunoglobulin (1 g/kg/day) was administered for two consecutive days for hypogammaglobulinemia (immunoglobulin G: 629 mg/dL).

The poor renal reserve precluded the patient from remdesivir therapy. Hydroxychloroquine with 400 mg twice daily was continued, and tocilizumab with a total dose of 400 mg (5.2 mg/kg) was prescribed 2 h after the initiation of CRRT. Tofacitinib (5 mg twice daily) was also prescribed for suspected antiphospholipid activity. We changed the AN69ST/PEA hemofilter circuit every 24 h for better clearance of cytokine/endotoxin. After 26-h treatment, the patient's hemodynamics improved. On day 20, we changed the CRRT hemofilter to an AN69 based hemofilter, after 3 days of cytokine adsorptive treatment. The viral load from the sputum was 2,762 copies/mL. Dopamine and norepinephrine were tapered off on day 22 (after 6-day cytokine-targeted therapy). The ferritin level declined from 13,317 ng/mL on day 16 to 3,875 ng/mL on day 21. The total bilirubin level was 2.28 mg/dL, and the triglyceride level decreased to 320 mg/dL. Another dose of tocilizumab with the same dosage of 400 mg was administered on day 25. The viral load at that time from sputum was 1.35 million copies/mL (Table 1).

**Table 1:** Baseline characteristics, treatment courses, and relevant data of the reported patient.

**Date (day of symptom onset)**	**1 April (11th)**	**5 April (16th)**	**13 April (24th)**	**22 April (33rd)**	**27 April (38th)**	**4 May (45th)**	**11 May (52nd)**
**Clinical status**							
BP (mmHg)	153/75	79/51	131/72	130/69	165/84	154/75	136/85
Pulse rates (bpm)		136	113	106	60	123	73
Urine output (mL/kg/h)	0	0.09	0.09	1.11	1.37	2.26	1.91
**Viral loads (copies/mL)**							
Sputum	Positive	2,762	1,354,580 (Day 26)	99.65	83.26	51.78	932.27
Throat swab	Positive	267	232,822				
Nasal swab			595,075 (Day 28)	72.46	1,128.65	6.59	65.67 (Day50)
**Vasopressors**							
Norepinephrine (mcg/kg/min)	–	0.26	0.02	0.01	–	–	–
Dopamine (mcg/kg/min)	–	3.70	1.45	0.00	–	–	–
VIS	0	29.80	2.95	1.40	0	0	0
**Hemogram**							
Hemoglobin (g/dL)	16.6	15.4	10.2	8.8	9.9	9.5	8.7
White blood cell (/μL)	4,900	12,430	19,430	11,480	6,610	13,180	11,660
Lymphocyte (/μL)		723	1,263	787	568	667 (D42)	1,118 (D51)
Lymphocyte (%)		6.1	6.5	7.4	8.6	8.5 (D42)	8.7 (D51)
Platelet (K/μL)	203	255	221	165	198	174	379
**Biochemistry**							
ALT (U/L)	44	47	27	18	25	89	107
AST(U/L)	45	121	83	47	40	57	58
BUN (mg/dL)	10	26.5	40	58.3	44.8	29.1	32.9
Creatinine (mg/dL)	1.12	3.1	2.7	4.1	1.6	1.9	1.1
Sodium (mmol/L)	–	144	133	137	137	142	138
Total Bilirubin (mg/dL)	1.1	2.18	1.39	1.76	2.43	4.76	2.77
CK (U/L)	261	820	1,672	288	128	107	219 (D49)
CK-MB (U/L)	–	8.53	7.35 (D23)	4.18	3.38 (D35)	3.14 (D42)	–
Ferritin (ng/mL)	–	13,317.09	3,993.02	2,461.61	1,636.28	2,690.1	3,200.13
Fibrinogen (mg/dL)	–	527.6 (D17)	363	240.5	227	233.1	178.2 (D53)
Lactic Acid (mmol/L)	–	5.83	1.44	2.54	0.9	0.94	0.77 (D51)
LDH (U/L)	–	813	663	412	358	427	302
TG (mg/dL)	–	446	320	116 (D34)	–	–	–
D-dimer (mg/L)	–	21.9	>35	>35	12.95	>35	6.81
**Inflammatory markers**							
Procalcitonin (ng/mL)	0.15	>100.0	4.03	2.15	0.587	0.645	0.128
CRP (mg/dL)	12.5	20.1	12.24	1.06	0.27	2.94	1.12
**Cytokines (pg/mL)**							
IL-6	–	12,049.1	3,546.5	2,535.4	154.0	1,579.02	
IL-4	–	0.46	0	0.66	0.07	0.1	
IL-10	–	2.45	2.22	5.24	3.71	4.77	
IL-17	–	0	0	0	0.55	0.88	
TNF	–	0	0	0	0	0	
IFN-gamma	–	0	0	0.03	0	2.01	
**Ventilator settings**							
Modes	Ambient Air	Pressure control	Pressure control	Pressure control			**Off on D52**
PaO_2_ (mmHg)		159	127	99	128	116	
FiO_2_		40%	40%	40%	40%	40%	
**CRRT settings**							
BFR (mL/min)		200	200	200	200	**Off on D40**	
Total wastes (mL/h)		2,000	2,000	2,000	1,700		
Anticoagulant		RCA	Heparin	Heparin	Heparin	
**VV-ECMO Settings**							
BFR (L/min)		3.7	3.75	3.3	3.91	2.58	**Off on D49**
ECMO FiO_2_		100%	50%	100%	40%	21%	
**Medications**							
Methylprednisolone (mg/kg/day)		–	–	0.4	0.4	0.3	0.4
Tocilizumab 400 mg			✓ (D25)			✓ (D49)	
Tofacitinib 5 mg BID		✓	✓				
Belimumab 400 mg						✓ (D49)	
**AN69ST/PEI**		**✓**		**✓**			

*COVID-19, coronavirus disease; ICU, intensive care unit; MV, mechanical ventilation; ECMO, extracorporeal membrane oxygenation; CRRT, continuous renal replacement therapy; AN69ST/PEI, hemofilter with AN69 and polyethyleneimine; BP, arterial blood pressure; VIS, vasoactive inotropic score; ALT, alanine aminotransferase; AST, aspartate aminotransferase; CK, creatine kinase; LDH, lactate dehydrogenase; BUN, blood urea nitrogen; CK, creatinine kinase; CK-MB, creatinine kinase muscle and brain form; CRP; TG, triglyceride; C-reactive protein; IL, interleukin; TNF, tumor necrotizing factor; IFN, interferon; FiO_2_, fraction of inhaled oxygen; VV-ECMO, veno-venous extracorporeal membrane oxygenation; BPF, blood flow rates.*

*✓: usage of this device or medications*.

On day 33, he was found to have an elevation of ferritin level and persistent tachycardia. Low dose norepinephrine (0.02 mcg/kg/min) was added. Ferritin levels increased from 1,761.15 to 2,535.39 ng/mL, and IL-6 levels increased from 1,477.59 to 2,535.39 pg/mL. There was persistent viral shedding from sputum with a viral load of 99.65 copies/mL. For suspected recurrence of CRS, another session of CRRT with an AN69ST/PEA hemofilter was performed on day 34, and the IL-6 level decreased to 707 pg/mL on day 35 and 154 pg/mL on day 38. We collected the CRRT effluent to estimate the removal of IL-6 from either CRRT or hemadsorption. The pre-AN69ST/PEI IL-6 level was 715.85 pg/mL, and the level 24 h later was 541.59 pg/mL. A total of 2,369,936 pg of IL-6 was removed, with 483,600 pg (20.4%) removed through the CRRT effluent and 1,886,336 pg (79.6%) removed by hemadsorptions. With adequate urine output and decreased oxygen demand, CRRT was discontinued on day 40.

He developed another recurrence of CRS on day 45 with an elevation of IL-6 (1,579 pg/mL) and ferritin (2,690 ng/mL) levels. An additional dose of tocilizumab 400 mg and belimumab (B-cell activating factor inhibitor) was administered. ECMO was terminated on day 49, and he was successfully extubated on day 52 (Figures 2B,C). The urine output was 2,000–2,500 mL/day after extubation. The urinalysis findings of dysmorphic RBC, glycosuria, leukocyturia, and RTC resolved. The follow-up diluted Russell viper venom test for lupus anticoagulant level was negative. The sputum RT-PCR revealed persistently positive results for SARS-CoV-2 until day 52. The patient was discharged without oxygen support on day 70 (Figure 1).

## Discussion

Critically ill COVID-19 patients, especially those with multi-organ failure, such as cardiovascular collapse, AKI, CRS, or thromboembolic events, challenge clinicians with a substantial risk of patient mortality (25–27) Thus, treatment for these patients should target multiple derangements induced by COVID-19, including ventilator support and ECMO for refractory hypoxemia, CRRT for AKI and extracorporeal cytokine removal, and biologic agents for cytokine blockade. We used a lung-protective strategy in mechanical ventilation and a CRRT with an AN69ST/PEA hemofilter for uremic toxin removal, volume management, cytokine, and endotoxin removal. VV-ECMO was initiated for oxygenation support. In addition, immunomodulating agents were prescribed to control CRS. We successfully treated a critically ill COVID-19 patient with predicted high mortality. This report also demonstrates a successful experience for managing severe COVID-19 without effective antiviral agents. We also recommend timely implementation of the cytokine-targeted strategy combining CRRT with an AN69ST/PEI hemofilter and tocilizumab for severe COVID-19 with CRS and multi-organ failure.

CRS in COVID-19 originates from a dysregulated immune response to SARS-CoV-2, which is not new to coronary virus infection (6, 28). Treatment for CRS includes removal of the offending pathogen and limiting the propagation of cytokines, in order to limit organ damage. Effective anti-SARS-CoV-2 therapies are not available at this time (29). Mechanical removal of the offending cytokines was reasonable. Methods for cytokine removal include direct hemoperfusion, conventional CRRT, high-dose CRRT, or CRRT with hemofilters with higher cut-off membranes (5). In patients with severe sepsis, CRRT with an AN69ST/PEA hemofilter might be associated with better outcomes. However, the only randomized control trial failed to balance its intervention group and placebo group and showed no benefit (16). However, CRRT with an AN69ST/PEA hemofilter has been shown to have better cytokine removal properties than conventional CRRT (16). This was also evident in our observation.

In addition to cytokine removal, we also used tocilizumab to neutralize the deleterious effects of IL-6. Recent publications have suggested that tocilizumab might mitigate CRS, although the studies were limited by non-controlled designs or case reports (30, 31) In their reports, the IL-6 level before tocilizumab treatment was 41 ng/L (interquartile range [IQR]: 10–102 ng/L), and elevated to 1,812 ng/L (IQR: 375–2,600 ng/L). Our case had a much higher pre-treatment level (12,049.07 ng/L); further, concurrent CRRT with an AN69ST/PEA hemofilter, the level rapidly reduced to 3,546.53 ng/L, and the second course of CRRT with an AN69ST/PEA hemofilter further reduced the IL-6 level from 2,535.39 ng/L to 139.39 ng/L. A retrospective study, based on data from 85 patients, demonstrated lower mortality rates. However, no IL-6 data were available in this study (32). We propose that there are synergistic benefits from combined CRRT with AN69ST/PEA hemofilter therapy and tocilizumab therapy.

Another concern about immunomodulation therapy in the absence of effective antiviral therapy is the potential for prolonged viral infection. In our patient, the viral shedding persisted for over 60 days. There is an urgent, but unmet, need for effective antiviral therapy in patients with severe COVID-19 and renal dysfunction. Remdesivir was not be considered universally to be an effective anti-viral agent for SARS-CoV-2; there were negative clinical trials, although the analytic methods may have led to negative trials (33). Of note, remdesivir is not indicated for patients with renal dysfunction (<30 mL/1.73 m^2^) (4).

VV-ECMO is a reasonable option for refractory ARDS caused by viral pneumonia. Based on the recent meta-analysis, the 90-day mortality rates decreased in the VV-ECMO arm (relative risk [RR] = 0.73 [95% CI 0.58–0.92]; *p* = 0.008) (34). But bleeding risk was one major complication for ECMO. The current guideline still use VV-ECMO as salvage therapy for severe ARDS with refractory hypoxemia. Advanced age, morbid obesity, and immunocompromised status are relative contra-indications as stated by ESLO guideline. It is not clear whether ECMO would be beneficial in patients with severe ARDS. But good organ support and meticulous prevention and management are keys to success in our patient (35). AKI is a common complication in patients with severe COVID-19 and is associated with worse outcomes (26). Interestingly, renal complications are not limited to AKI; hematuria, proteinuria, or glycosuria also develop with COVID-19 (9). Evidence has shown that hematuria, proteinuria, and glycosuria develop during the critical illness and subside after renal recovery, similar to that observed in our patient (36). We had limited renal biopsy data to explore the underlying pathogenesis of SARS-CoV-2 infections (37), although data from SARS-CoV may give us hints that renal damage from COVID-19 is diverse (38). The increased hematuria and proteinuria are also associated with adverse outcomes (36).

The plain radiograph is less sensitive than chest CT, but chest radiography is typically the first-line imaging modality ordered for patients with suspected COVID-19. Compared to typical bacteremic pneumonia, the correlation of chest radiography to oxygen demand is poor. In our patient, more consolidation was detected on chest radiography when less O_2_ demand, shown in Figure 2. The follow-up chest radiograph revealed sequelae of pulmonary fibrosis. It is not useful to predict severity by image evaluation once before diagnosis or during treatment for COVID-19 (39).

There were some limitations to this case report. As a report of a single case, it is not possible to generalize the experience to the whole patient group with severe COVID-19. However, we are confident that this report would inspire future clinical trials involving the cytokine-targeted therapy, proposed in this study. Second, the patient was treated in an area where there were few critical cases and thus, cannot be generalized to a medical system overwhelmed by the COVID-19 pandemic (2). However, if further clinical trials prove its efficacy, timely cytokine-targeted therapy for critically ill patients with COVID-19 is likely to improve patient outcomes. Third, we did not use antiviral therapy for this patient. Poor renal reserve precluded the patient from remdesivir therapy (4, 29).

In summary, we demonstrated in this report that timely combined hemoperfusion with cytokine adsorptive capacity and anti-cytokine therapy may successfully treat COVID-19 patients with devastating CRS. For the rapid progression of severe COVID-19 ARDS, cytokine releasing syndrome, acute kidney injury, and multiple organ failure. We would suggest timely initiation of life support and use of multimodality treatment for blockade of cytokine effects (by tocilizumab) and rapid removal of pro-inflammatory cytokines to limit organ damage. This further highlights the importance of implementing cytokine-targeted therapy in severe COVID-19 guided by precise measurement of disease activity.

## Data Availability Statement

The raw data supporting the conclusions of this article will be made available by the authors, without undue reservation.

## Ethics Statement

Written informed consent was obtained from the individual(s) for the publication of any potentially identifiable images or data included in this article.

## Author Contributions

TH, Y-CC, C-HW, S-YC, B-LC, and S-CH performed the data collection. TH, Y-CC, C-HW, Y-CY, S-CK, and AT wrote the manuscript. J-TW, C-JY, and S-CC carried out the project administration. All authors contributed to the article and approved the submitted version.

## Conflict of Interest

The authors declare that the research was conducted in the absence of any commercial or financial relationships that could be construed as a potential conflict of interest.

## Abbreviations

ALT: alanine aminotransferase
AKI: acute kidney injury
APACHE II: Physiologic Assessment and Chronic Health Evaluation II
AST: aspartate aminotransferase
RBC: red blood cell
RTC: renal tubular cells
CBA: cytometric bead array
COVID-19: coronavirus disease
CRP: C-reactive protein
CRRT: continuous renal replacement therapy
CRS: cytokine release syndrome
GFR: glomerular filtration rate
ICU: intensive care unit
IL: interleukin
RCA: regional citrate anticoagulation
RT-PCR: real-time polymerase chain reaction
SARS-CoV-2: severe acute respiratory syndrome coronavirus 2
SOFA: sequential organ failure assessment
VIE: Vasoactive-Inotropic Equivalent
VV-ECMO: veno-venous extracorporeal membrane oxygenation.
